# Identification of key biomarkers and therapeutic targets in sepsis through coagulation-related gene expression and immune pathway analysis

**DOI:** 10.3389/fimmu.2024.1470842

**Published:** 2024-10-04

**Authors:** Jing Ge, Qijie Deng, Rui Zhou, Yahui Hu, Xiaotong Zhang, Zemao Zheng

**Affiliations:** ^1^ Department of Pediatrics, Nanfang Hospital, Southern Medical University, Guangzhou, Guangdong, China; ^2^ Grade 2020, The First Clinical Medical School, Southern Medical University, Guangzhou, Guangdong, China; ^3^ Department of Oncology, Nanfang Hospital, Southern Medical University, Guangzhou, Guangdong, China; ^4^ Guangdong Province Key Laboratory of Molecular Tumor Pathology, Guangzhou, Guangdong, China; ^5^ Department of Huiqiao Medical Centre, Nanfang Hospital, Southern Medical University, Guangzhou, Guangdong, China; ^6^ Department of Ultrasound, Shandong Provincial Third Hospital, Shandong University, Jinan, Shandong, China; ^7^ Department of Respiratory and Critical Care Medicine, Nanfang Hospital, Southern Medical University, Guangzhou, Guangdong, China

**Keywords:** sepsis, coagulation-related genes, immune response, FCER1G, FYN

## Abstract

Sepsis, characterized by a widespread and dysregulated immune response to infection leading to organ dysfunction, presents significant challenges in diagnosis and treatment. In this study, we investigated 203 coagulation-related genes in sepsis patients to explore their roles in the disease. Through differential gene expression analysis, we identified 20 genes with altered expression patterns. Subsequent correlation analysis, visualized through circos plots and heatmaps, revealed significant relationships among these genes. Gene Ontology (GO) and Kyoto Encyclopedia of Genes and Genomes (KEGG) pathway enrichment analyses indicated that these genes are involved in immune response activation, coagulation, and immune receptor activity. Disease Ontology (DO) enrichment analysis further linked these genes to autoimmune hemolytic anemia and tumor-related signaling pathways. Additionally, the CIBERSORT analysis highlighted differences in immune cell composition in sepsis patients, revealing an increase in neutrophils and monocytes and a decrease in inactive NK cells, CD8 T cells, and B cells. We employed machine learning techniques, including random forest and SVM, to construct a diagnostic model, identifying FCER1G and FYN as key biomarkers. These biomarkers were validated through their expression levels and ROC curve analysis in an independent validation cohort, demonstrating strong diagnostic potential. Single-cell analysis from the GSE167363 dataset further confirmed the distinct expression profiles of these genes across various cell types, with FCER1G predominantly expressed in monocytes, NK cells, and platelets, and FYN in CD4+ T cells and NK cells. Enrichment analysis via GSEA and ssGSEA revealed that these genes are involved in critical pathways, including intestinal immune networks, fatty acid synthesis, and antigen processing. In conclusion, our comprehensive analysis identifies FCER1G and FYN as promising biomarkers for sepsis, providing valuable insights into the molecular mechanisms of this complex condition. These findings offer new avenues for the development of targeted diagnostic and therapeutic strategies in sepsis management.

## Introduction

Sepsis is a serious worldwide health issue marked by a strong, systemic response to infection that results in organ dysfunction (1–3). Sepsis continues to be the world’s biggest cause of death in intensive care units despite advancements in medical care (4, 5). The complexity of sepsis, marked by its heterogeneous etiology and variable clinical presentation, poses significant challenges in its diagnosis and management (6–8). Traditionally, sepsis was understood primarily as a disorder of systemic inflammation (9). However, recent insights have revealed that it is a more complex syndrome involving various aspects of the immune response, coagulation pathways, and cellular metabolism (10).

The dysregulated immune response is one of the central features of sepsis (11, 12). Initially, there is an overwhelming pro-inflammatory response aimed at controlling the infection, often followed by a compensatory anti-inflammatory response (13–15). This biphasic pattern can lead to immune paralysis, making patients susceptible to secondary infections (16). In order to create effective therapeutic strategies, it is essential to comprehend the mechanisms behind this dysregulated immune response.

The coagulation system plays a critical role (17–19). The cross-talk between inflammation and coagulation pathways exacerbates the severity of sepsis (20, 21). A high mortality rate is associated with the advancement of disseminated intravascular coagulation (DIC) in several septic patients (22, 23). However, the relationship between specific coagulation-related genes and the onset and progression of sepsis is still inadequately understood.

The current criteria for diagnosis of sepsis are based on clinical signs and biomarkers such as procalcitonin (PCT) and C-reactive protein (CRP), which are not unique to sepsis and differ widely among individuals (24, 25). Moreover, the therapeutic strategies are mainly supportive, focusing on infection control and organ support rather than targeting the underlying pathophysiological mechanisms of sepsis (9, 26).

Given the challenges of diagnosing and treating sepsis, there is an urgent need to deepen our understanding of its molecular and cellular mechanisms. Identifying genetic markers and pathways related to coagulation and immune response may provide crucial insights into sepsis’s pathophysiology, leading to more targeted therapeutic interventions and diagnostic tools that could improve patient outcomes (27, 28). This study addresses these gaps by investigating the link between sepsis and coagulation-related genes. We hypothesize that specific genes within the coagulation cascade play critical roles in the onset and progression of sepsis, with their expression patterns potentially serving as diagnostic markers or therapeutic targets. Utilizing advanced bioinformatics and machine learning techniques, we comprehensively examined these genes in sepsis patients, aiming to uncover the genetic basis of sepsis and pave the way for more personalized and effective management of this complex condition.

## Methods

### Data collection

The sepsis patient dataset GSE85233, comprising 22 normal and 51 sepsis samples, was retrieved from the Gene Expression Omnibus (GEO) database. Additionally, single-cell RNA sequencing data were obtained from the GEO dataset GSE167363. For independent validation, another dataset, GSE57065, including 25 control and 28 sepsis samples, was utilized.

### Gene selection

A curated list of 203 coagulation-related genes was compiled using gene sets from the Gene Set Enrichment Analysis (GSEA) database. These gene sets were derived from the pathways hsa04610 and hsa04611, which are associated with coagulation and related processes.

### Differential gene expression analysis

The differential expression analysis of coagulation-related genes in sepsis patients was performed using the ‘limma’ package in R. To visualize the results, a volcano plot was generated with ‘ggpubr,’ and a heatmap depicting the expression levels of differentially expressed genes was created using the ‘heatmap’ package.

### Correlation analysis

A correlation analysis examined the interrelationship among the 20 differently expressed genes. A correlation heatmap was generated in R using the “corrplot” package, and a circos plot created using the “RCircos” package was used to visualize the results (29).

### Functional enrichment analysis

Gene Ontology (GO) and Disease Ontology (DO) enrichment analysis was performed using the ‘clusterProfiler’ package to identify the biological functions and disease associations of the differentially expressed genes (30). Additionally, a Kyoto Encyclopedia of Genes and Genomes (KEGG) pathway analysis was carried out using “clusterProfiler” to investigate the roles of genes in different biological pathways (31).

### Chromosomal location analysis

Using the “RCircos” package, which shows genomic data in a circular form and enables the identification of potential chromosomal patterns related to changes in gene expression in sepsis, the chromosomal positions of the 20 differentially expressed coagulation-related genes were determined.

### Machine learning for diagnostic model construction

A diagnostic model was constructed using machine learning techniques. The ‘randomForest’ package was used for random forest analysis to determine the key genes differentiating sepsis patients from controls. Support Vector Machine (SVM) analysis was conducted with the ‘SVM’ function within the ‘e1071’ package to optimize the accuracy and minimize error based on feature selection. The ‘glmnet’ package was utilized for lasso analysis to find diagnostic biomarkers by applying a penalty to the coefficient sizes. The common key genes among the different machine learning approaches were identified through a Venn diagram using the ‘Venn’ package.

### Validation of key gene expression and ROC curves

The ‘limma’ package, intended for gene expression data analysis from microarray or RNA-seq technologies, validated the key gene expression. The ‘timeROC’ package was utilized to perform Receiver Operating Characteristic (ROC) curve analysis to evaluate the identified biomarkers’ diagnostic efficacy.

### Biological function and pathway enrichment analysis

The ‘patchwork’ and ‘org.Hs.eg.db’ packages were used to conduct Gene Set Enrichment Analysis (GSEA) to investigate the key genes’ biological function and pathway enrichment analysis. Single-sample GSEA (ssGSEA) was used to identify important signaling pathways differently activated in sepsis patients compared to healthy controls. The analysis was done using ‘Limma’, and the correlation between the key genes and the signaling pathways was shown using ‘ggplot2’.

### Single-Cell RNA Sequencing Analysis

Following quality control filtering of single-cell RNA sequencing data from the GSE167363 dataset, the remaining cells were processed for dimensionality reduction and clustering. The ‘SingleR’ package was utilized for automated cell-type annotation, while ‘ggplot2’ was employed to assess the expression levels of key genes in different cell types.

### Immune cell deconvolution

The ‘CIBERSORT’ package, based on gene expression profiles and a predefined signature matrix of immune cell types, was used to deconvolute the immune cell proportions in sepsis patients. We visualized the differences in immune cell fractions between the control and sepsis samples using the ‘ggpubr’ package.

### PCA and clustering for subtype analysis

Based on the patterns of gene expression, different sepsis subtypes were distinguished using Principal Component Analysis (PCA) utilizing the ‘prcomp’ and ‘ggplot2’ functions. To classify sepsis patients into distinct subtypes, consensus clustering was carried out with the help of the ‘ConsensusClusterPlus’ package (32, 33). The ‘pheatmap’ package was used to create heatmaps of gene expression, and ‘ggpubr’ was used to generate box plots for visual comparison of differential gene expression across subtypes.

### RNA extraction and qRT-PCR analysis

This study was approved by the Ethics Committee. Five sepsis patients and five healthy individuals undergoing routine health examinations were recruited at Nanfang Hospital, Southern Medical University, between December 2023 and January 2024. Peripheral blood mononuclear cells (PBMCs) were isolated from patients’ peripheral blood samples using previously described methods (34). Total RNA was extracted from PBMC samples using the FastPure Cell/Tissue Total RNA Isolation Kit (Vazyme). RNA was then reverse-transcribed into cDNA using the ReverTra Ace qPCR RT Master Mix and gDNA Remover Kit. Quantitative real-time PCR (qRT-PCR) was performed using the SYBR Premix Ex Taq II in a real-time fluorescence quantitative PCR system, with GAPDH selected as the endogenous control for mRNA. The reaction conditions were as follows: initial denaturation at 95°C for 10 minutes, followed by 45 cycles of 95°C for 5 seconds and 60°C for 30 seconds (35). The amplification of target genes and internal reference genes was performed separately for each sample, with each group of samples containing three replicate wells. Data analysis was conducted using the 2^(-ΔΔCt) method. The primer sequences are provided in **Supplementary File 1**.

### Statistical analysis

Rstudio was used for all statistical analysis and computational modeling (version 4.2.1).

## Results

### Analysis of differential gene expression and correlation in coagulation-related genes associated with sepsis

**Figure 1:**
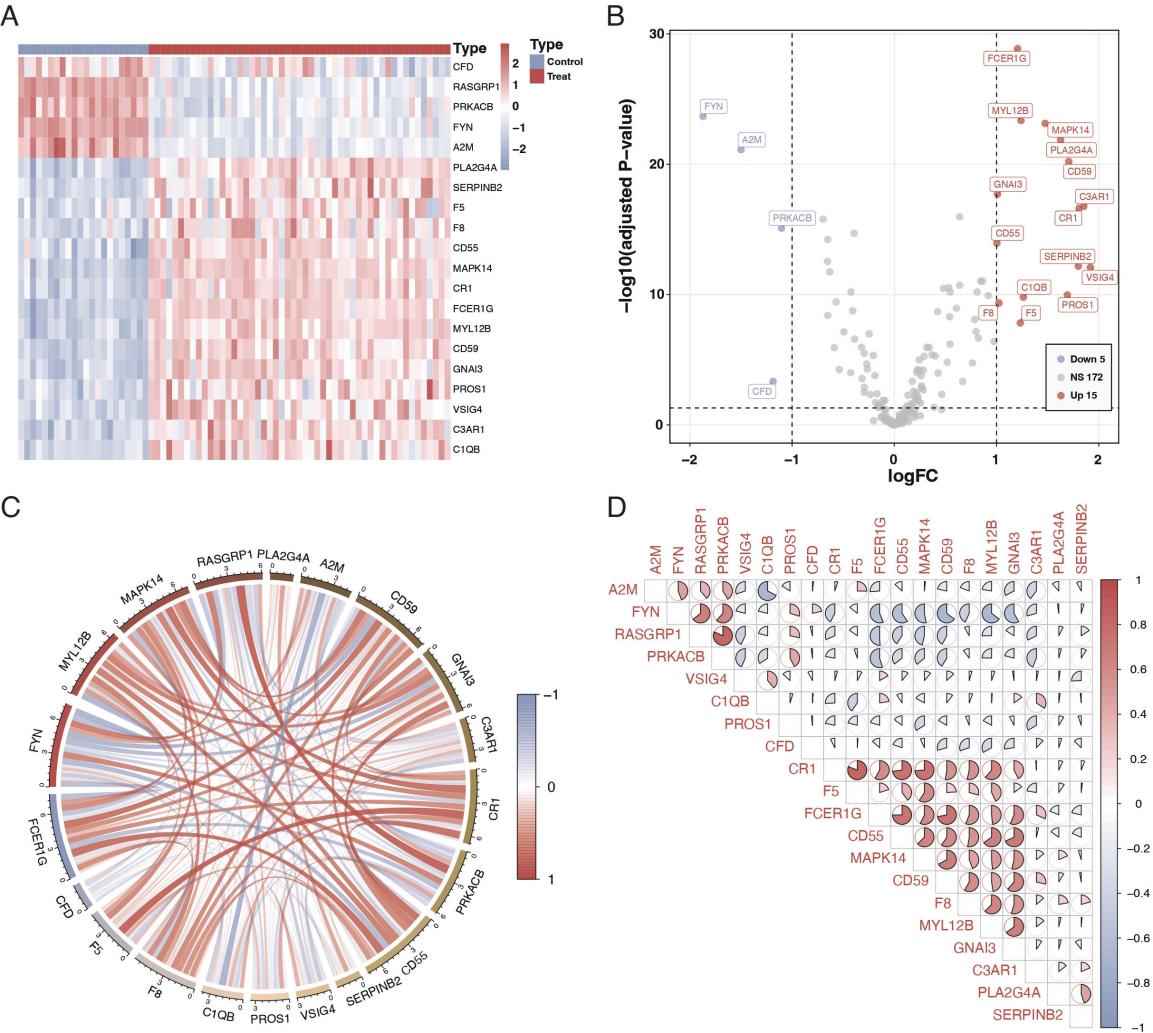
Differential expression and correlation of coagulation-related genes in sepsis. **(A)** Heatmap illustrating the differential expression of 20 coagulation-related genes between sepsis patients and controls. Each row represents a gene, and each column represents a sample. Red indicates higher expression, and blue indicates lower expression relative to the mean. **(B)** Volcano plot showing the differential expression of coagulation-related genes. The x-axis represents the log fold change, and the y-axis represents the negative logarithm of the p-value. Genes marked in red are significantly upregulated, green represents downregulated genes, and gray indicates no significant difference. **(C)** Circos plot depicting the correlation among the 20 differentially expressed genes. The circle segments represent individual genes, and the connecting ribbons indicate the correlation strength (red for positive and green for negative correlation). **(D)** Correlation matrix displaying the pairwise correlations between the 20 differentially expressed genes. Red circles indicate positive correlations, and green circles indicate negative correlations, with the color’s intensity corresponding to the correlation’s strength.

We studied 203 coagulation genes using differential gene expression analysis to comprehend the connection between coagulation-related genes and sepsis. This analysis revealed 20 genes that exhibited significant differential expression, as shown in **Figures 1A, B**. Furthermore, we conducted a correlation study to explore the relationships among these genes. The results from the circos plot indicated a substantial correlation among all 20 genes, as depicted in **Figure 1C**. Additionally, the heatmap of correlations reinforced these findings, demonstrating a close association between these 20 genes, as illustrated in **Figure 1D**.

### Gene ontology and pathway enrichment analysis of differentially expressed genes

**Figure 2:**
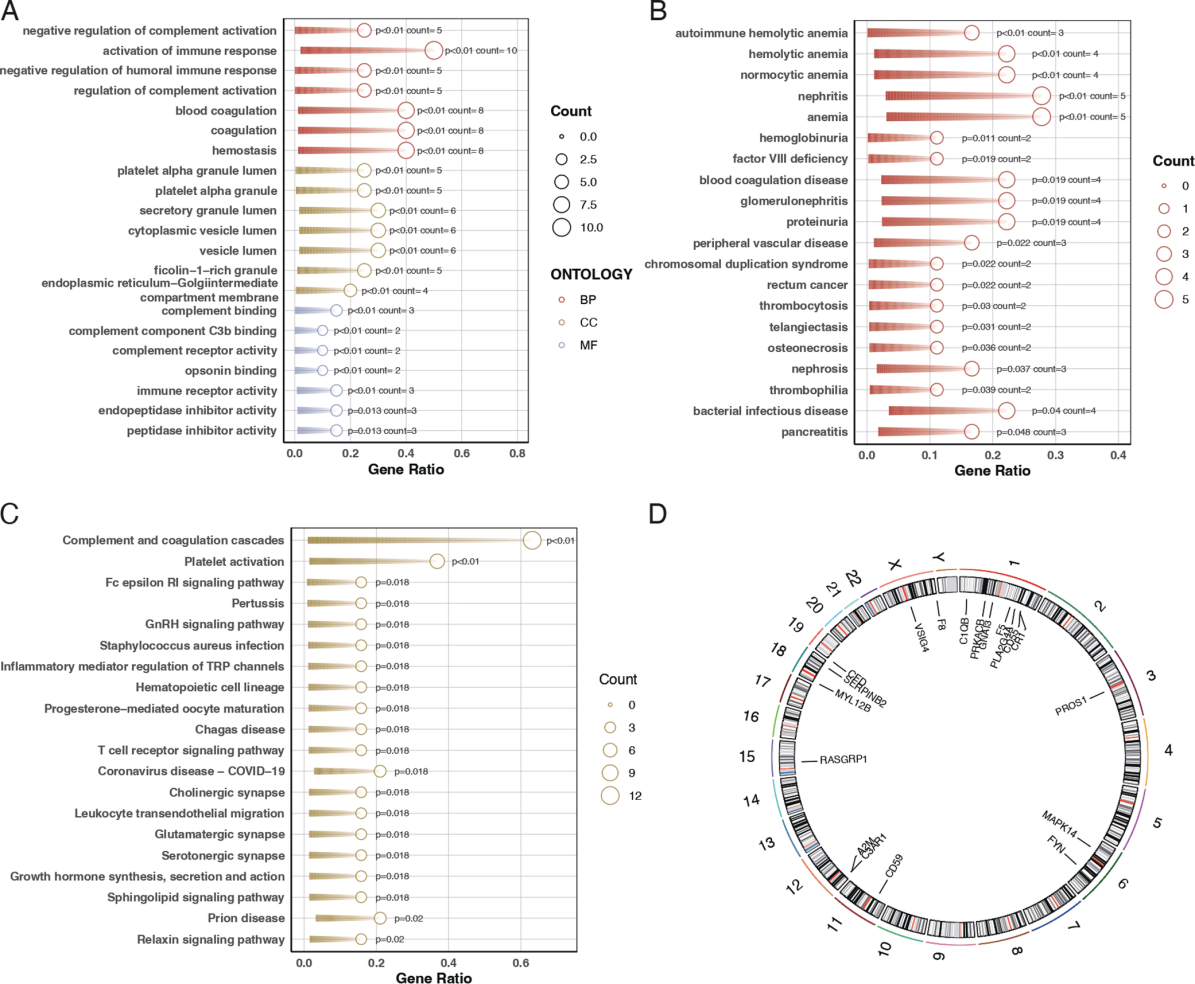
Gene ontology and pathway enrichment analysis. **(A)** Bubble chart for Gene Ontology (GO) analysis showing the biological functions associated with the differentially expressed genes. The bubble size represents the gene count, and the color indicates the p-value, with darker shades representing higher significance. **(B)** Bar plot of Disease Ontology (DO) enrichment analysis indicating diseases and conditions related to the differentially expressed genes. Red bars represent conditions with the highest gene counts, and purple bars indicate conditions with lower counts. **(C)** Bar plot of KEGG pathway enrichment analysis showing the pathways in which the differentially expressed genes are involved. Red bars represent pathways with the highest gene counts, and purple bars indicate pathways with lower counts. **(D)** Circos plot illustrating the chromosomal distribution of the 20 differentially expressed genes. Each gene is positioned according to its location on the chromosome.

To elucidate the biological roles of the differentially expressed genes, we conducted Gene Ontology (GO) analysis and pathway enrichment analysis using the Kyoto Encyclopedia of Genes and Genomes (KEGG). The GO analysis indicated that these genes are primarily involved in biological functions such as immune receptor activity, coagulation, secretory granule lumen composition, and immune response activation (**Figure 2A**). The Disease Ontology (DO) enrichment analysis revealed that these genes are enriched in pathways related to tumor signaling and autoimmune hemolytic anemia (**Figure 2B**). KEGG pathway analysis demonstrated that these differentially expressed genes are mainly concentrated in pathways associated with complement system activation and platelet activation (**Figure 2C**). Additionally, the chromosomal locations of these 20 genes were visualized using a circos plot (**Figure 2D**).

### Immune cell differential in sepsis patients

**Figure 3:**
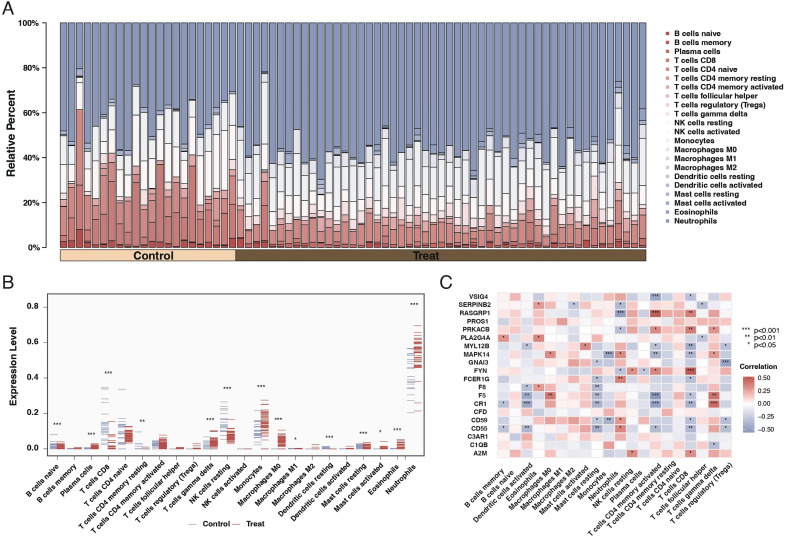
Immune cell composition and gene correlation in sepsis. **(A)** Stacked bar chart showing the relative proportion of different immune cell types in control and sepsis patient samples. Each color represents a different cell type, with the height of the color indicating the cell type’s relative abundance. **(B)** Dot plot comparing the fraction of immune cell types between control and sepsis patient samples. The x-axis lists the cell types, and the y-axis shows the fraction of each cell type, with blue dots representing controls and red dots representing sepsis patients. Asterisks indicate significance levels. **(C)** Correlation heatmap of the 20 differentially expressed genes against different immune cell types. The color and size of each square indicate the correlation coefficient, with red for positive and blue for negative correlations. Asterisks denote significance.

Our results indicated a significant relationship between sepsis and immune responses. Further investigation through CIBERSORT analysis elucidated the differences in immune cell composition in sepsis patients. The findings revealed that sepsis patients exhibited lower levels of unactivated B cells, CD8 T cells, and unactivated NK killer cells while having higher proportions of monocytes and neutrophils, as shown in **Figures 3A, B**. Subsequent correlation analysis between the 20 differentially expressed genes and immune cells demonstrated that most genes significantly associate with immune cells, as depicted in **Figure 3C**.

### Subtyping of sepsis patients based on differential gene expression

**Figure 4:**
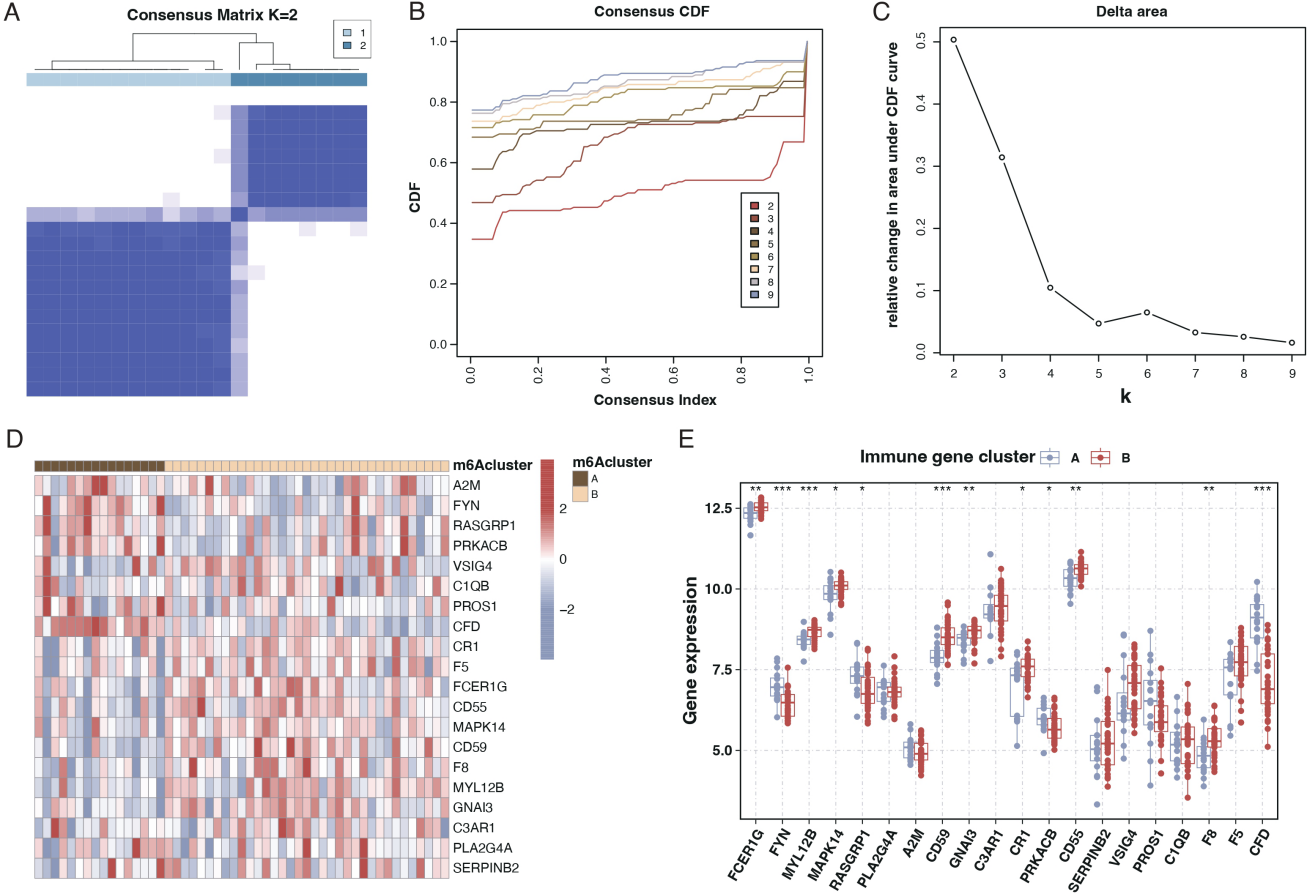
Subtyping of sepsis patients and differential gene expression. **(A)** Consensus matrix heatmap from consensus clustering showing two distinct clusters (k=2) of sepsis patients based on differential gene expression profiles. **(B)** A Consensus Cumulative Distribution Function (CDF) plot is used to determine the number of clusters (k), each color representing a different k value. **(C)** Delta area plot depicting the relative change in area under the CDF curve for each k, aiding in selecting the optimal number of clusters. **(D)** Heatmap displaying the expression patterns of the 20 differentially expressed genes across the two identified sepsis subtypes. Genes and patient subtypes are clustered based on expression similarity. **(E)** Boxplots illustrating the expression levels of the 20 differentially expressed genes in the two sepsis patient subtypes, with significant differences denoted by asterisks.

We employed consensus clustering through differential gene expression analysis to categorize sepsis patients into two subtypes, as illustrated in **Figures 4A–C**. Analysis of the differential genes between these two subtypes revealed that most of the 20 genes exhibited significant differences, as presented in **Figures 4D, E**.

### PCA and immune cell infiltration analysis in sepsis subtypes

**Figure 5:**
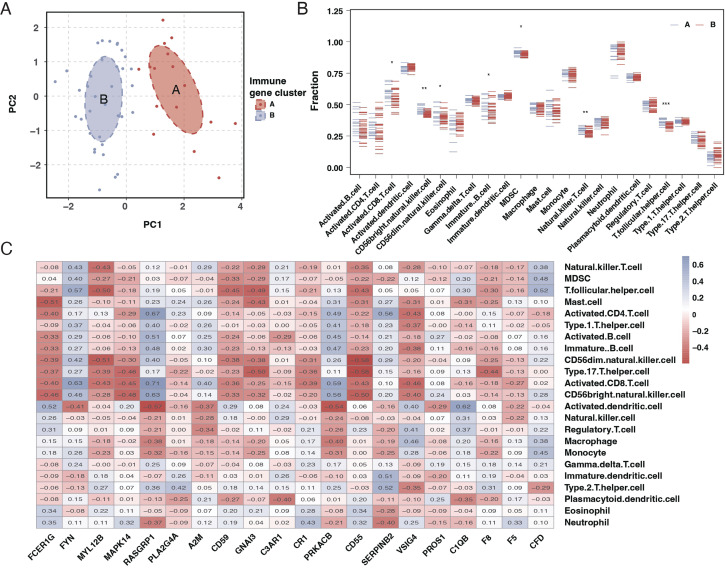
PCA and immune cell infiltration analysis in sepsis subtypes. **(A)** PCA plot showing the separation of the two sepsis subtypes, with each subtype forming a distinct cluster. **(B)** Boxplots comparing the immune cell infiltration profiles between the two sepsis subtypes across various immune cell types. **(C)** Heatmap of the correlation matrix between the 20 differentially expressed genes and the immune cell types, with colors indicating the strength and direction of correlation. * mean P < 0.05, ** mean P < 0.01, *** mean P < 0.001.

Principal Component Analysis (PCA) results suggested a good distinction between the two sepsis subtypes, as indicated in **Figure 5A**. An examination of immune cell infiltration in these subtypes revealed that subtype A had higher immune cell infiltration, as shown in **Figure 5B**. A heatmap of correlations highlighted significant relationships between immune cells and the 20 genes, as seen in **Figure 5C**.

### Development and validation of a diagnostic model using machine learning

**Figure 6:**
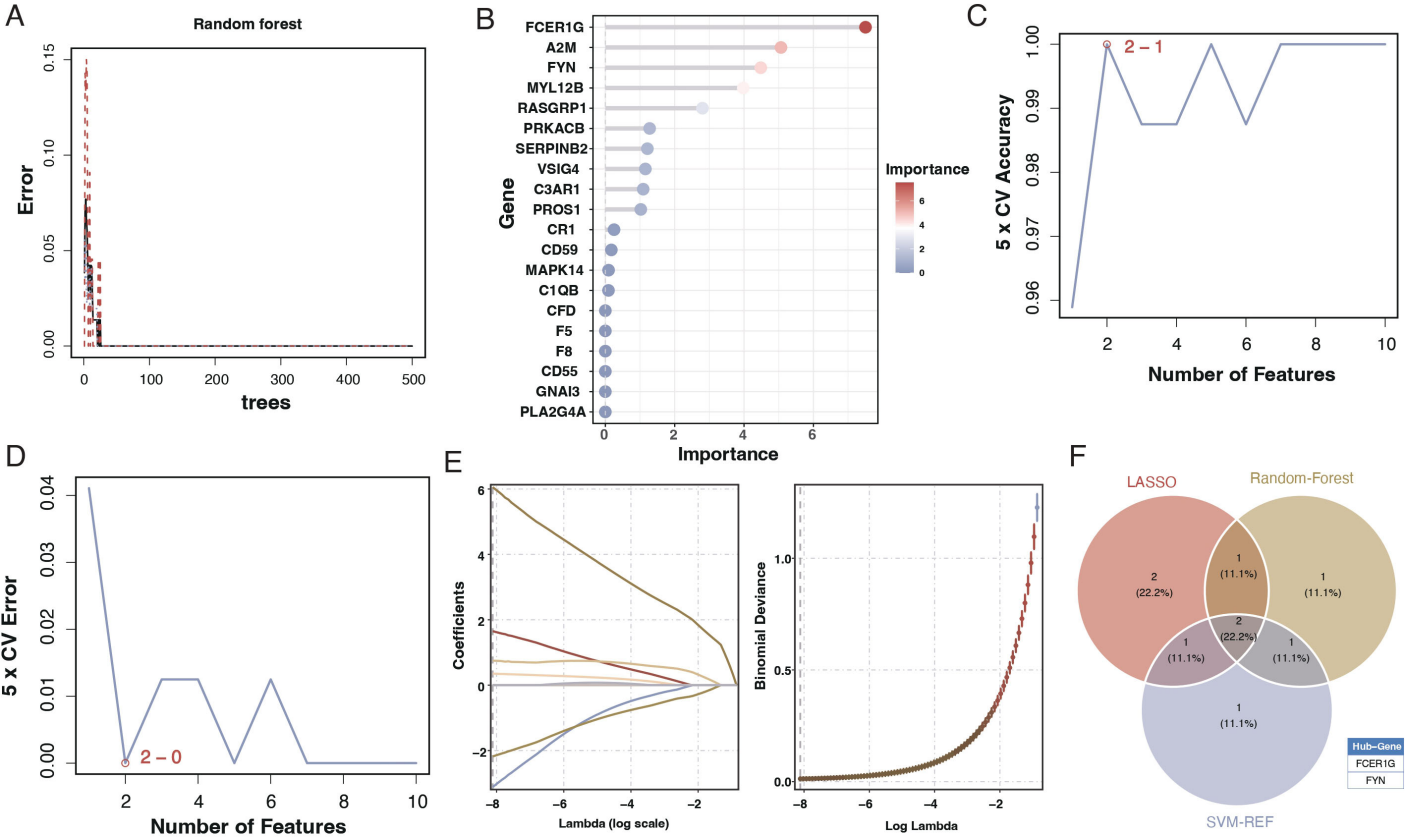
Machine learning analysis for diagnostic model development. **(A)** Random forest analysis showing the error rate as a function of the number of trees used in the model. **(B)** Variable importance plot from the random forest model, with the size and color of the dots representing the importance of each gene in the model. **(C)** Line graph displaying the SVM model’s 5-fold cross-validation (CV) accuracy as the number of features varies. **(D)** Line graph showing the 5-fold CV error rate from the SVM model for different numbers of features. €: Lasso coefficient profile plot against the log(lambda) sequence, with vertical lines drawn at the optimal values using cross-validation. **(F)** Venn diagram depicting the intersection of key genes identified as potential biomarkers from the random forest, SVM, and Lasso analyses.

**Figure 7:**
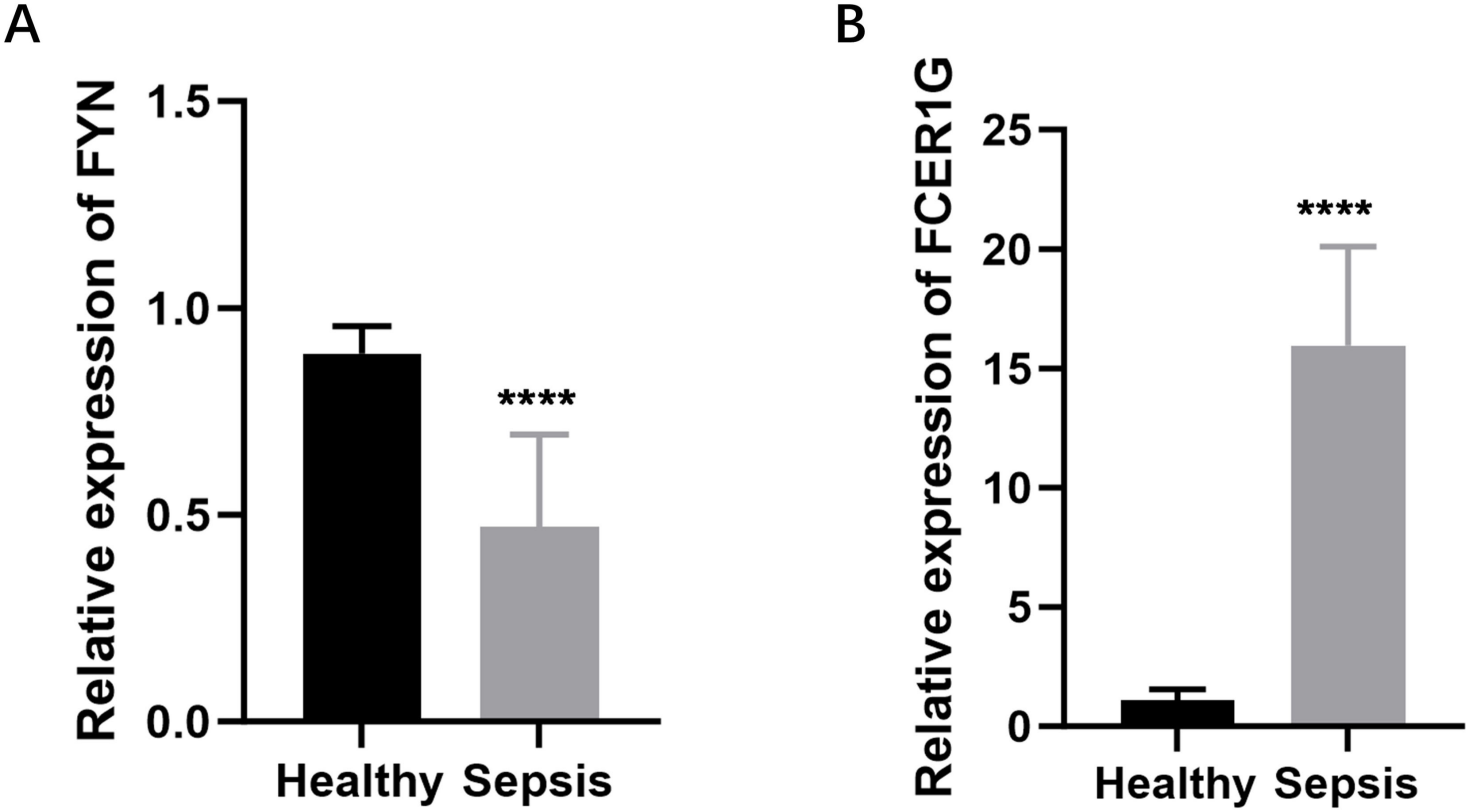
Expression levels of FYN and FCER1G in sepsis patients and healthy controls. **(A)** The relative expression of FYN in peripheral blood mononuclear cells (PBMCs) from sepsis patients compared to healthy controls. **(B)** The relative expression of FCER1G in PBMCs from sepsis patients compared to healthy controls. **** P < 0.0001.

To create a diagnostic model, we used machine learning techniques. As shown in **Figures 6A, B**, random forest analysis identified the top five key genes, including FCER1G and A2M. The Support Vector Machine (SVM) results indicated that the selection of two genes offered the best accuracy and minimal error. However, we chose a set of five genes for further analysis, achieving an accuracy of 1 and an error rate of 0, as depicted in **Figures 6C, D**. Lasso analysis identified six diagnostic biomarkers, as shown in **Figure 6E**. To develop a common key gene diagnostic model, a Venn diagram was utilized to find the intersecting genes among the three analyses, resulting in two intersecting genes: FYN and FCER1G, as illustrated in **Figure 6F**. The qRT-PCR results showed that FYN was downregulated in the sepsis patient group compared to the healthy control group (**Figure 7A**), while FCER1G was upregulated in the sepsis patient group compared to the healthy control group (**Figure 7B**).

### Diagnostic efficacy of the identified biomarkers

**Figure 8:**
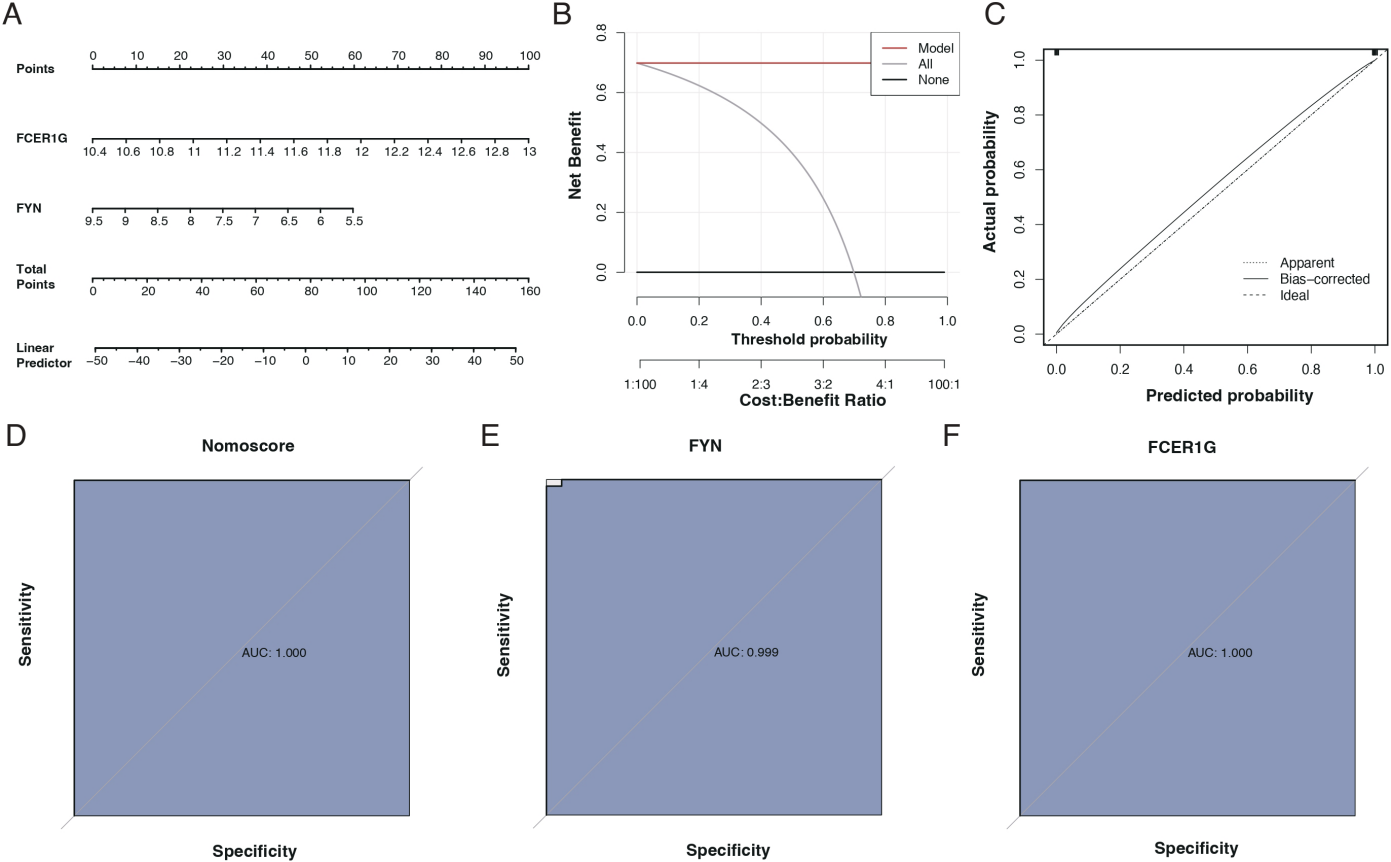
Diagnostic performance of biomarkers FCER1G and FYN. **(A)** Nomogram for sepsis prediction, incorporating the biomarkers FCER1G and FYN. The top scale represents the total points calculated by summing the assigned points for each biomarker, correlating with the probability of sepsis. **(B)** Decision Curve Analysis (DCA) showing the net benefit of using the nomogram across different threshold probabilities. **(C)** Calibration curve of the nomogram. The diagonal dotted line represents a perfect prediction by an ideal model. The solid line represents the performance of the nomogram, with the closer fit to the diagonal dotted line indicating better prediction. **(D)** Receiver Operating Characteristic (ROC) curve for the nomogram. The AUC of 1.000 suggests the perfect discriminative ability of the nomogram for predicting sepsis. **(E)** ROC curve for the biomarker FYN with an AUC of 0.999, indicating near-perfect diagnostic performance. **(F)** ROC curve for the biomarker FCER1G with an AUC of 1.000, indicating perfect diagnostic performance.

We assessed the diagnostic capability of the two identified biomarkers through column line graphs, as seen in **Figure 8A**. Decision Curve Analysis (DCA) suggested that patients could benefit from these biomarkers, as shown in **Figure 8B**. Calibration curves indicated a minimal difference between the actual risk of sepsis and the predicted risk, signifying the high accuracy of the model, as presented in **Figure 8C**. The ROC curves of the column line graphs demonstrated good predictive performance, as depicted in **Figure 8D**. Additionally, the diagnostic ROC for the key genes, FYN and FCER1G, showed areas under the curve (AUC) of 0.999 and 1.000, respectively, indicating excellent diagnostic efficacy, as shown in **Figures 8E, F**.

### Biological function analysis of key genes

**Figure 9:**
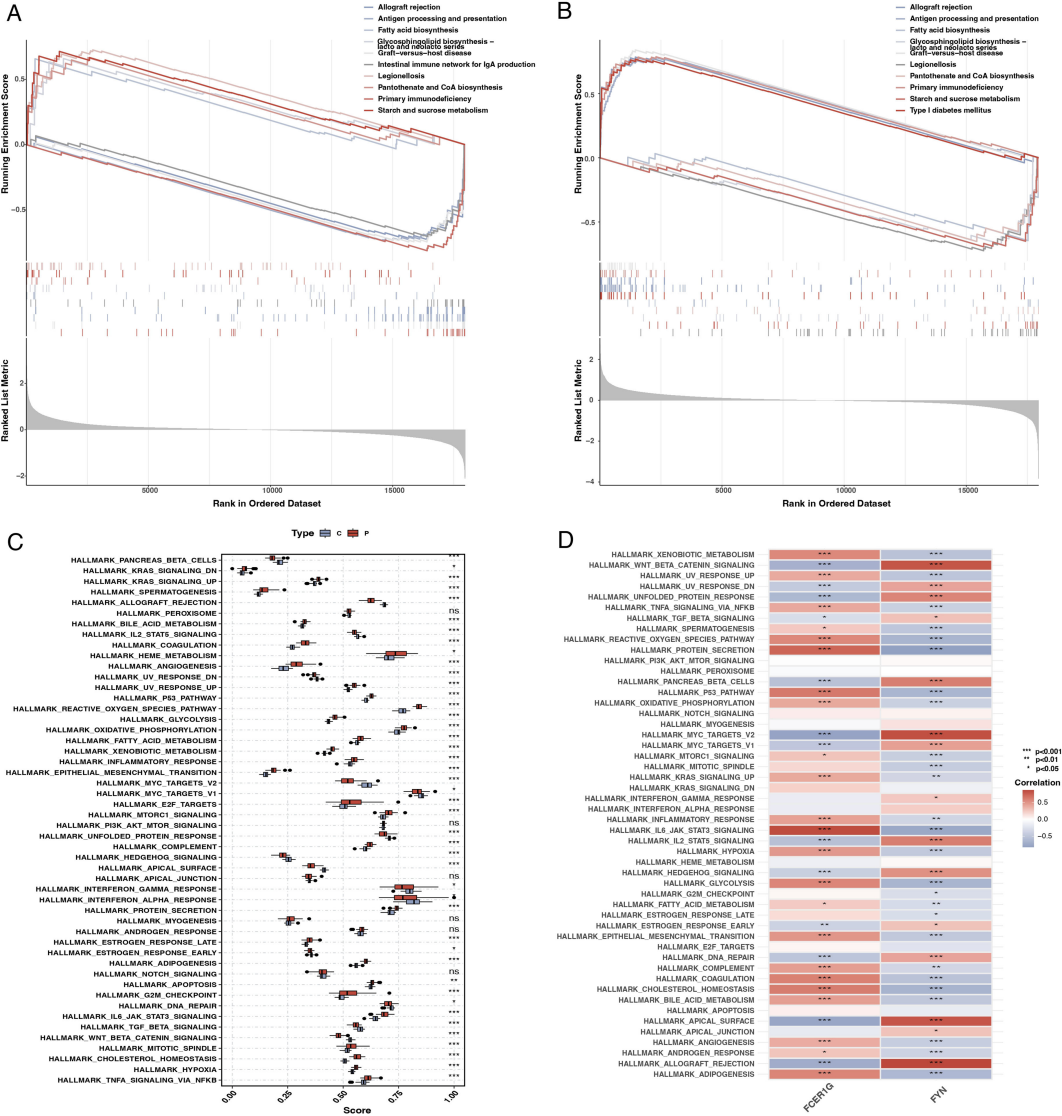
Gene set enrichment analysis (GSEA) and single-sample GSEA (ssGSEA). **(A)** GSEA plot for FCER1G showing the enrichment of gene sets across the ranked list of genes in the dataset, with pathways related to fatty acid synthesis and IgA production significantly enriched. **(B)** GSEA plot for FYN highlighting the enriched gene sets, including those related to transplant rejection and antigen processing. **(C)** Box plot representing ssGSEA scores for different hallmark pathways in sepsis patients and controls, indicating significant upregulation of specific pathways in sepsis. **(D)** Heatmap showing the correlation of FCER1G and FYN expression with the ssGSEA scores for the hallmark pathways, illustrating significant associations with most pathways.

GSEA enrichment analysis revealed that FCER1G was predominantly associated with pathways such as fatty acid synthesis and the intestinal immune network for IgA production (**Figure 9A**). In contrast, FYN was primarily enriched in pathways related to transplant rejection, antigen processing, and presentation (**Figure 9B**). Using ssGSEA, we examined differences between sepsis patients and healthy controls across 50 hallmark signaling pathways. In sepsis patients, several hallmark pathways, including KRAS_SIGNALING_DNE, REACTIVE_OXYGEN_SPECIES_PATHWAY, and TNFA_SIGNALING_VIA_NFKB, were significantly upregulated (**Figure 9C**). Additionally, a correlation analysis between the two key genes and various hallmark pathways revealed significant associations with most pathways (**Figure 9D**).

### Validation of key genes and diagnostic model efficacy

**Figure 10:**
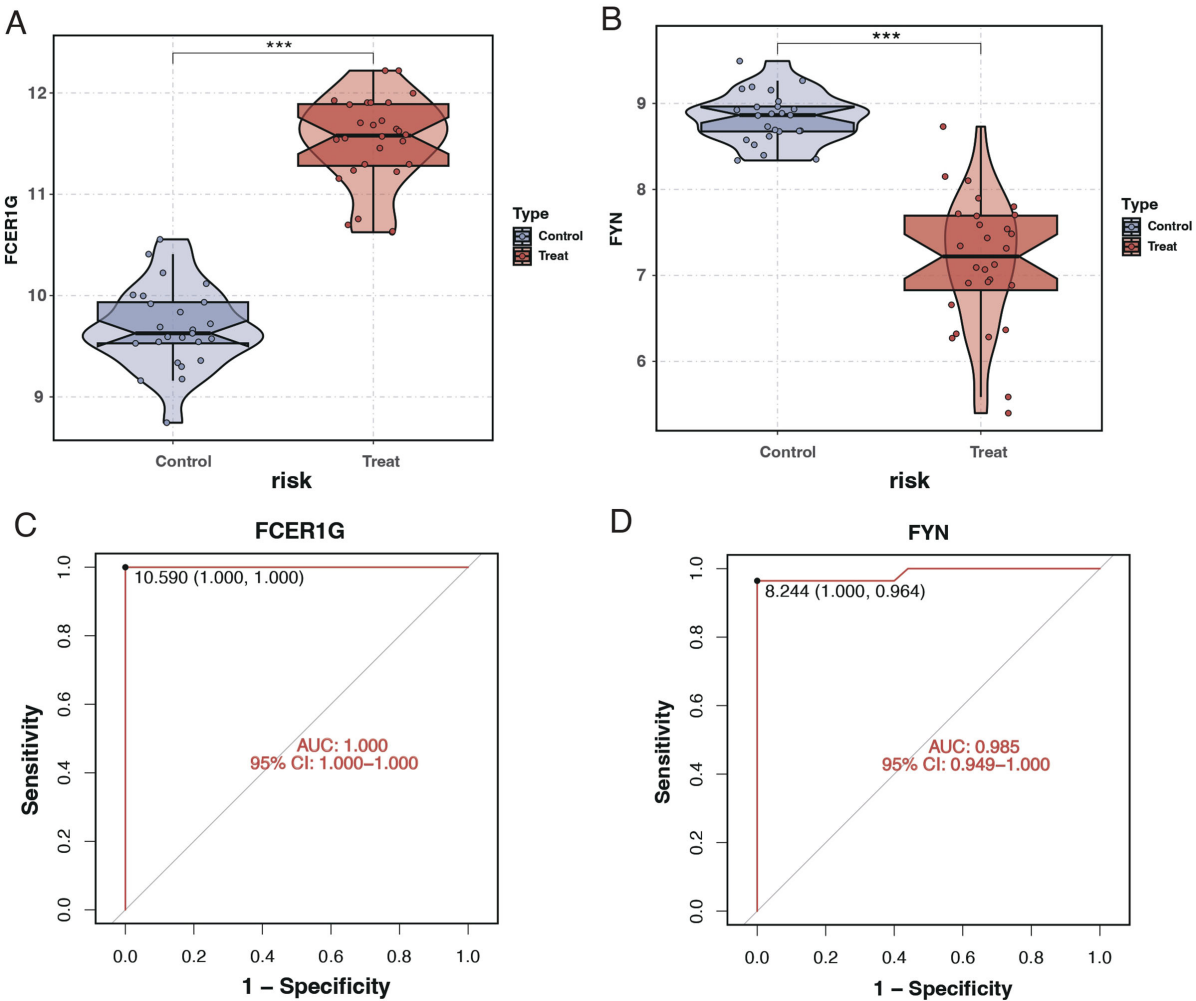
Expression analysis and ROC curves of FCER1G and FYN in validation cohort. **(A)** Violin plot displaying the expression levels of FCER1G in the control and treatment groups, with significant overexpression in sepsis patients. **(B)** Violin plot showing the expression levels of FYN in the control and treatment groups, with significant underexpression in sepsis patients. **(C)** ROC curve for FCER1G in the validation cohort, with the AUC indicating excellent diagnostic accuracy. **(D)** The ROC curve for FYN in the validation cohort also shows high diagnostic accuracy with an AUC of 0.985. *** P < 0.001.

The expression levels of the key genes and their diagnostic efficacy were validated in a separate cohort. FCER1G expression was found to be elevated in sepsis patients (**Figure 10A**), while FYN expression was reduced (**Figure 10B**), consistent with findings from the training set. ROC curve analysis demonstrated the strong diagnostic potential of these genes, with the area under the curve (AUC) being 1 for FCER1G and 0.985 for FYN (**Figures 10C, D**).

### Single-cell analysis from the GSE167363 dataset

**Figure 11:**
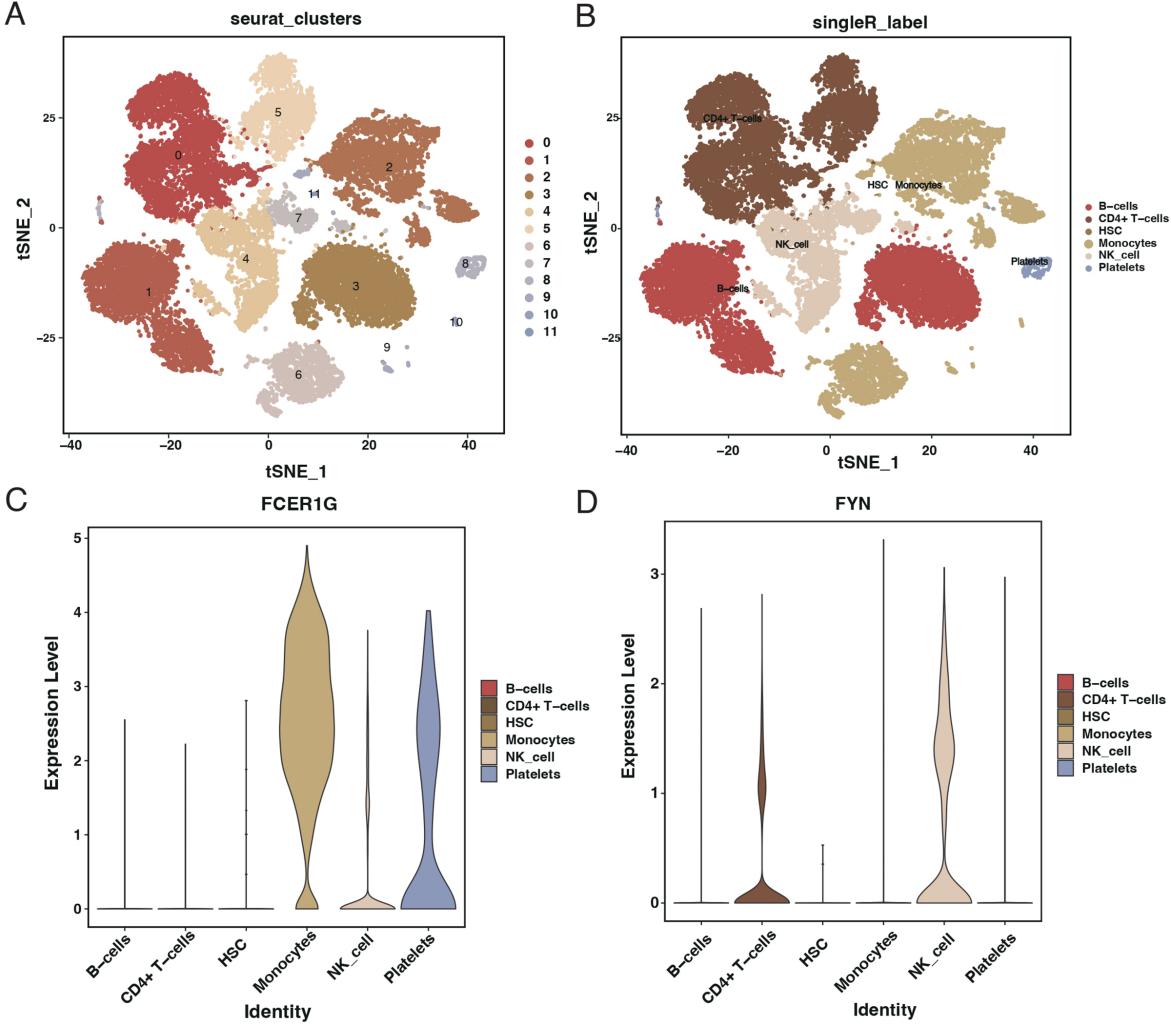
Single-cell RNA sequencing analysis from GSE167363 dataset. **(A)** t-SNE plot illustrating the clustering of single cells from the GSE167363 dataset into 11 distinct groups. **(B)** t-SNE plot with cell types annotated, identifying six major cell populations within the dataset. **(C)** Violin plots showing the expression level of FCER1G across different identified cell types, with higher expression in monocytes, NK cells, and platelets. **(D)** Violin plots depicting the expression level of FYN across various cell types, with higher expression in CD4+ T cells and NK cells.

After quality control filtering, 25,458 cells were extracted from the GSE167363 dataset. Post-dimensionality reduction and clustering, these cells were categorized into 11 distinct clusters (**Figure 11A**). Further cell annotation identified six cell types (**Figure 11B**). FCER1G was found to be highly expressed in monocytes, NK cells, and platelets (**Figure 11C**), whereas FYN showed higher expression in CD4+ T cells and NK cells (**Figure 11D**).

## Discussion

This study embarked on an exploration of the complex interplay between coagulation-related genes and sepsis, utilizing a combination of differential gene expression analysis, machine learning, and pathway enrichment techniques. Our findings revealed significant alterations in the expression of several coagulation-related genes in sepsis patients, with FCER1G and FYN emerging as potential biomarkers. The robustness of these biomarkers was validated through various analytical methods, including random forest, SVM, and ROC curve analyses.

Identifying FCER1G and FYN as key players in sepsis aligns with and extends the findings of previous research (36). For instance, studies have highlighted the role of FCER1G in immune regulation and its potential as a biomarker in other inflammatory diseases (37). Our findings corroborate these studies and further illuminate their significance in sepsis. Similarly, FYN, known for its role in T-cell signaling and immune responses, has been implicated in other pathological conditions, but its specific role in sepsis has been less clear (38). Our research bridges this gap, providing evidence of its involvement in sepsis pathogenesis.

Patients with sepsis exhibit higher expression of FCER1G and lower expression of FYN, suggesting a complex interplay between these genes in the immune response during sepsis. The roles these genes play in the immune system, particularly in modulating inflammation and immune cell activation, are crucial for understanding the pathophysiology of sepsis. Our findings indicate that these genes are essential in the body’s response to infection and developing sepsis. These pathways include fatty acid synthesis, the intestinal immune network’s ability to produce IgA, and the processing and presentation of antigens.

Our study’s insights into FCER1G and FYN enhance our understanding of sepsis and open new avenues for diagnostics and therapeutics. The high diagnostic accuracy of these genes, as indicated by their AUC values, underscores their potential as biomarkers for early detection of sepsis. Furthermore, understanding their role in sepsis pathophysiology could lead to the development of targeted therapies, which could be more effective than the current broad-spectrum approaches.

## Limitations

While our study offers significant new insights, it has several limitations. The generalizability of our findings may be influenced by the diversity of the patient population and the sample size. Future research should focus on larger and more diverse cohort studies to validate our results. Additionally, experimental studies are needed to fully understand the mechanisms by which FCER1G and FYN influence sepsis progression. This could include both *in vitro* and *in vivo* experiments to elucidate the molecular pathways involved and assess their potential as therapeutic

## Conclusion

In conclusion, our study illuminates the complex genetic landscape of sepsis, with a particular focus on the roles of FCER1G and FYN. The identification of these genes as potential diagnostic markers and therapeutic targets offers promise for developing improved management strategies for sepsis, a condition that continues to pose significant challenges in critical care. Integrating our findings with ongoing research into sepsis pathophysiology has the potential to revolutionize our understanding and treatment of this life-threatening condition.

## Data Availability

The datasets presented in this study can be found in online repositories. The names of the repository/repositories and accession number(s) can be found in the article/**Supplementary Material**.

## Supplementary material

Supplementary File 1 Primer sequences used for qRT-PCR analysis.

## References

[1] Ackerman MH Ahrens T Kelly J Pontillo A. Sepsis. Crit Care Nurs Clinics North America. (2021) 33:407–18. doi: 10.1016/j.cnc.2021.08.003 34742497

[2] Gotts JE Matthay MA. Sepsis: pathophysiology and clinical management. Bmj. (2016) 353:i1585. doi: 10.1136/bmj.i1585 27217054

[3] Vincent JL. Current sepsis therapeutics. EBioMedicine. (2022) 86:104318. doi: 10.1016/j.ebiom.2022.104318 36470828 PMC9782815

[4] Gauer R Forbes D Boyer N. Sepsis: diagnosis and management. Am Family Phys. (2020) 101:409–18. 32227831

[5] Labib A. Sepsis care pathway 2019. Qatar Med J. (2019) 2019:4. 10.5339/qmj.2019.qccc.4 PMC6851952 31763206

[6] Sinha P Meyer NJ Calfee CS. Biological phenotyping in sepsis and acute respiratory distress syndrome. Annu Rev Med. (2023) 74:457–71. doi: 10.1146/annurev-med-043021-014005 PMC10617629 36469902

[7] Stanski NL Wong HR. Prognostic and predictive enrichment in sepsis. Nat Rev Nephrol. (2020) 16:20–31. doi: 10.1038/s41581-019-0199-3 31511662 PMC7097452

[8] Wang W Liu CF. Sepsis heterogeneity. World J Pedia: WJP. (2023) 19:919–27. doi: 10.1007/s12519-023-00689-8 36735197

[9] Jarczak D Kluge S Nierhaus A. Sepsis-pathophysiology and therapeutic concepts. Front Med. (2021) 8:628302. doi: 10.3389/fmed.2021.628302 PMC8160230 34055825

[10] Giustozzi M Ehrlinder H Bongiovanni D Borovac JA Guerreiro RA Gąsecka A. Coagulopathy and sepsis: Pathophysiology, clinical manifestations and treatment. Blood Rev. (2021) 50:100864. doi: 10.1016/j.blre.2021.100864 34217531

[11] Wiersinga WJ van der Poll T. Immunopathophysiology of human sepsis. EBioMedicine. (2022) 86:104363. doi: 10.1016/j.ebiom.2022.104363 36470832 PMC9783164

[12] Zhang YY Ning BT. Signaling pathways and intervention therapies in sepsis. Signal Transduct Target Ther. (2021) 6:407. doi: 10.1038/s41392-021-00816-9 34824200 PMC8613465

[13] Maneta E Aivalioti E Tual-Chalot S Emini Veseli B Gatsiou A Stamatelopoulos K. Endothelial dysfunction and immunothrombosis in sepsis. Front Immunol. (2023) 14:1144229. doi: 10.3389/fimmu.2023.1144229 37081895 PMC10110956

[14] van der Poll T Shankar-Hari M Wiersinga WJ. The immunology of sepsis. Immunity. (2021) 54:2450–64. doi: 10.1016/j.immuni.2021.10.012 34758337

[15] Luo H Li Y Song H Zhao K Li W Hong H. Role of EZH2-mediated epigenetic modification on vascular smooth muscle in cardiovascular diseases: A mini-review. Front Pharmacol. (2024) 15:1416992. doi: 10.3389/fphar.2024.1416992 38994197 PMC11236572

[16] Téblick A Gunst J Langouche L Van den Berghe G. Novel insights in endocrine and metabolic pathways in sepsis and gaps for future research. Clin Sci (Lond). (2022) 136:861–78. doi: 10.1042/CS20211003 35642779

[17] Arina P Singer M. Pathophysiology of sepsis. Curr Opin Anaesthesiol. (2021) 34:77–84. doi: 10.1097/ACO.0000000000000963 33652454

[18] Iba T Helms J Connors JM Levy JH. The pathophysiology, diagnosis, and management of sepsis-associated disseminated intravascular coagulation. J Intensive Care. (2023) 11:24. doi: 10.1186/s40560-023-00672-5 37221630 PMC10202753

[19] Iba T Levy JH. Sepsis-induced coagulopathy and disseminated intravascular coagulation. Anesthesiology. (2020) 132:1238–45. doi: 10.1097/ALN.0000000000003122 32044801

[20] Iba T Levi M Levy JH. Intracellular communication and immunothrombosis in sepsis. J Thromb Haemost: JTH. (2022) 20:2475–84. doi: 10.1111/jth.15852 PMC9804233 35979601

[21] Tsantes AG Parastatidou S Tsantes EA Bonova E Tsante KA Mantzios PG. Sepsis-induced coagulopathy: an update on pathophysiology, biomarkers, and current guidelines. Life. (2023) 13. doi: 10.3390/life13020350 PMC9961497 36836706

[22] Iba T Connors JM Nagaoka I Levy JH. Recent advances in the research and management of sepsis-associated DIC. Int J Hematol. (2021) 113:24–33. doi: 10.1007/s12185-020-03053-y 33386597 PMC7775827

[23] Rinaldi I Sudaryo MK Prihartono NA. Disseminated intravascular coagulation in sepsis and associated factors. J Clin Med. (2022) 11. doi: 10.3390/jcm11216480 PMC9658286 36362708

[24] Barichello T Generoso JS Singer M Dal-Pizzol F. Biomarkers for sepsis: more than just fever and leukocytosis-a narrative review. Crit Care (London England). (2022) 26:14. doi: 10.1186/s13054-021-03862-5 PMC8740483 34991675

[25] Komorowski M Green A Tatham KC Seymour C Antcliffe D. Sepsis biomarkers and diagnostic tools with a focus on machine learning. EBioMedicine. (2022) 86:104394. doi: 10.1016/j.ebiom.2022.104394 36470834 PMC9783125

[26] Evans L Rhodes A Alhazzani W Antonelli M Coopersmith CM French C. Surviving sepsis campaign: international guidelines for management of sepsis and septic shock 2021. Crit Care Med. (2021) 49:e1063–e143. 10.1097/CCM.0000000000005337 34605781

[27] Jacobi J. Pathophysiology of sepsis. Am J health-system pharm: AJHP. (2002) 59 Suppl 1:S3–8. doi: 10.1093/ajhp/59.suppl_1.S3 11885412

[28] Zhang P Zou B Liou YC Huang C. The pathogenesis and diagnosis of sepsis post burn injury. Burns Trauma. (2021) 9:tkaa047. doi: 10.1093/burnst/tkaa047 33654698 PMC7901709

[29] Zhang H Meltzer P Davis S. RCircos: an R package for Circos 2D track plots. BMC Bioinf. (2013) 14:244. doi: 10.1186/1471-2105-14-244 PMC3765848 23937229

[30] Wu T Hu E Xu S Chen M Guo P Dai Z. clusterProfiler 4.0: A universal enrichment tool for interpreting omics data. Innovation (Cambridge (Mass)). (2021) 2:100141. doi: 10.1016/j.xinn.2021.100141 34557778 PMC8454663

[31] Zhao K Wu X Han G Sun L Zheng C Hou H. Phyllostachys nigra (Lodd. ex Lindl.) derived polysaccharide with enhanced glycolipid metabolism regulation and mice gut microbiome. Int J Biol Macromol. (2024) 257:128588. doi: 10.1016/j.ijbiomac.2023.128588 38048922

[32] Wilkerson MD Hayes DN. ConsensusClusterPlus: a class discovery tool with confidence assessments and item tracking. Bioinformatics. (2010) 26:1572–3. doi: 10.1093/bioinformatics/btq170 PMC2881355 20427518

[33] Li Q Qi L Zhao K Ke W Li T Xia L. Integrative quantitative and qualitative analysis for the quality evaluation and monitoring of Danshen medicines from different sources using HPLC-DAD and NIR combined with chemometrics. Front Plant Sci. (2022) 13:932855. doi: 10.3389/fpls.2022.932855 36325569 PMC9618615

[34] Zhou J Liu J Zhang C Zhou Y Zheng Z Li H. Elucidating the molecular mechanisms of sepsis: Identifying key aging-related biomarkers and potential therapeutic targets in the treatment of sepsis. Environ Toxicol. (2024). doi: 10.1002/tox.24198 38440848

[35] Wang J An G Peng X Zhong F Zhao K Qi L. Effects of three Huanglian-derived polysaccharides on the gut microbiome and fecal metabolome of high-fat diet/streptozocin-induced type 2 diabetes mice. Int J Biol Macromol. (2024) 273:133060. doi: 10.1016/j.ijbiomac.2024.133060 38871107

[36] Gong FC Ji R Wang YM Yang ZT Chen Y Mao EQ. Identification of potential biomarkers and immune features of sepsis using bioinformatics analysis. Mediators Inflamm. (2020) 2020:3432587. doi: 10.1155/2020/3432587 33132754 PMC7568774

[37] Yu Y Li J Li J Zen X Fu Q. Evidence from machine learning, diagnostic hub genes in sepsis and diagnostic models based on xgboost models, novel molecular models for the diagnosis of sepsis. Curr Med Chem. (2023). doi: 10.2174/0109298673273009231017061448 37921181

[38] Wang H Huang J Yi W Li J He N Kang L. Identification of immune-related key genes as potential diagnostic biomarkers of sepsis in children. J Inflamm Res. (2022) 15:2441–59. doi: 10.2147/JIR.S359908 PMC9015049 35444449

