# Supplementary material for: Identification of key biomarkers and therapeutic targets in sepsis through coagulation-related gene expression and immune pathway analysis
Source: Front Immunol. 2024 Oct 4;15:1470842. doi: 10.3389/fimmu.2024.1470842 (PMC11486639; doi:10.3389/fimmu.2024.1470842)
Supplement: Supplementary File 1 — Primer sequences used for qRT-PCR analysis. [file Table1.docx]

**Supplementary table 1. A list of primers used in this study.**

| Gene | Forward sequence (5’ to 3’) | Reverse sequence (5’ to 3’) |
| --- | --- | --- |
| GAPDH | GGAGCGAGATCCCTCCAAAAT | GGCTGTTGTCATACTTCTCATGG |
| FCER1G | AGCAGTGGTCTTGCTCTTACT | TGCCTTTCGCACTTGGATCTT |
| FYN | ACTACCCCAGCTTCGGTGT | CTCCAAAGACGGTGAGTCCT |
